# Clinical implication of three-dimensional heart models for planning of surgical procedure in complex congenital heart disease

**DOI:** 10.1186/1532-429X-11-S1-P275

**Published:** 2009-01-28

**Authors:** Eugenie Riesenkampff, Urte Rietdorf, Ivo Wolf, Bernhard Schnackenburg, Michael Hübler, Vladimir Alexi-Meskishvili, Hans-Peter Meinzer, Roland Hetzer, Felix Berger, Titus Kuehne

**Affiliations:** 1 German Heart Institute Berlin, Berlin, Germany; 2 German Cancer Research Center Heidelberg, Heidelberg, Germany; 3 Deutsches Krebsforschungszentrum Heidelberg, Heidelberg, Germany; 4 Philips Medical Systems, Hamburg, Germany

**Keywords:** Conventional Magnetic Resonance Imaging, Heart Model, Optimum Treatment Strategy, Conventional Magnetic Resonance, Complex Congenital Heart Disease

## Background and aims

In patients with complex congenital heart diseases detailed three-dimensional information about intra- and extracardiac anatomy are essential for planning of surgical treatment strategies. However, it is a reality that current standard imaging techniques and their post-processing methods do not always provide the means to elucidate all required anatomic information in a comprehensive manner. We report our initial experiences with the use of realistic three-dimensional models of the heart in the clinical setting.

## Methods

In 11 patients (age 0.7 – 27 years) with complex congenital heart disease, surgical questions were directed towards palliative or corrective surgery but consensus about the optimum treatment strategy was not reached using standard diagnostic tools including echocardiography, catheterization and conventional magnetic resonance imaging (MRI). In these patients, three-dimensional printed cast and virtual computer models of the heart were made on the basis of high-resolution whole-heart and/or cineMRI. Anatomic descriptions were compared with intraoperative findings when surgery was performed.

## Results

MRI provided, independent from age related factors, sufficient image quality for producing the heart models. In all patients, the models clearly evidenced the anatomy in an unequivocal three-dimensional context. This was, as an adding to standard diagnostic imaging, invaluable information that supported surgical decision making. The anatomy as shown in the models was confirmed during surgery. Biventricular corrective surgery was planned in five patients, palliative surgery in three patients and in another 3 patients no option for surgery was seen.

## Conclusion

Realistic three-dimensional models of the heart provide a new means for the assessment of complex anatomy. This method can facilitate surgical decision making and preoperative planning.

